# Thyroglossal duct lipoma: A rare case report

**DOI:** 10.1097/MD.0000000000020392

**Published:** 2020-05-29

**Authors:** Ming-Shao Tsai, Geng-He Chang, Huei-Chieh Chuang, Wan-Ni Lin, Yao-Te Tsai

**Affiliations:** a Department of Otolaryngology – Head and Neck Surgery, Chiayi Chang Gung Memorial Hospital; b Health Information and Epidemiology Laboratory of Chiayi Chang Gung Memorial Hospital, Chiayi; c Graduate Institute of Clinical Medical Sciences, Chang Gung University; d Faculty of Medicine, College of Medicine, Chang Gung University, Taoyuan; e Department of Pathology, Chiayi Chang Gung Memorial Hospital, Chiayi; f Department of Otolaryngology – Head and Neck Surgery, Linkou Chang Gung Memorial Hospital, Taoyuan, Taiwan.

**Keywords:** adipose, lipoma, thyroglossal duct cyst

## Abstract

**Rationale:**

Thyroglossal duct cyst (TGDC), the most common midline neck mass, has several histological diagnoses other than cyst in the literature. We present the first case of thyroglossal duct lipoma.

**Patient concerns:**

A 63-year-old woman presented with a painless soft midline neck mass for more than 3 years, which enlarged in size and caused a lump sensation during swallowing.

**Diagnoses:**

Sonography revealed a 3.5 × 3.0 × 3.0-cm^3^ homogenous isoechoic oval lesion without an acoustic shadow beyond the thyroid glands. An ultrasound-guided biopsy revealed abundant sheets of fat cells with infiltration of some lymphocytes and histiocytes. Computed tomography revealed a 3.5 × 3.0 × 3.0-cm^3^ well-circumscribed ovoid mass with Hounsfield unit (HU) between −50 and −100 and a thyroglossal duct remnant. All these findings supported the diagnosis of thyroglossal duct lipoma.

**Interventions:**

The patient underwent Sistrunk operation for excision of the neck tumor, and pathological examination revealed an adipose tumor surrounded by benign thyroid tissue, confirming the diagnosis of thyroglossal duct lipoma.

**Outcomes:**

Neither postoperative complication nor recurrence was noted at the 18-month follow-up.

**Lessons:**

This is the first case of thyroglossal duct lipoma in the literature. Our study extends the disease spectrum of thyroglossal duct mass and suggests that clinicians should consider thyroglossal duct lipoma in the differential diagnosis of a midline neck mass.

## 1 Introduction

Thyroglossal duct cyst (TGDC), the most common diagnosis for midline neck mass, is usually located between the thyroid glands and hyoid bone; however, it may present in any location between the tongue base and suprasternal regions due to the embryologic pathogenesis.^[1,2]^ Histopathological diagnoses other than cyst have been reported for TGDC and include thyroid goiter, aberrant lymph node, and malignancy.^[1,3]^ However, TGDC comprising lipoma has never been reported. We report the first case of thyroglossal duct lipoma.

## 2 Case report

The Institutional Review Board of Chang Gung Memorial Hospital approved this study (CGMH-IRB No.101-4516B). Patient has provided informed consent for publication of the case.

**Figure 1:**
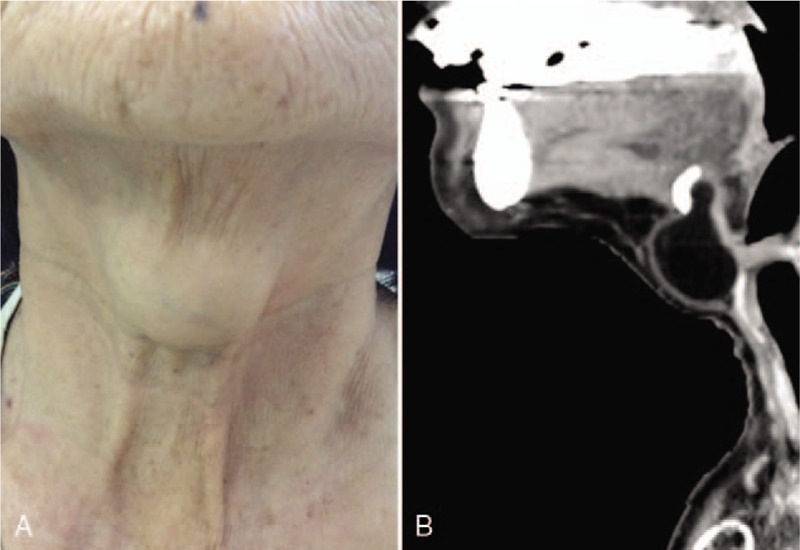
A 63-year-old female patient with thyroglossal duct lipoma. (A) Photograph of the patient presenting a midline neck mass (grossly 3.5 × 3.0 cm^2^) located between the thyroid cartilage and hyoid bone. (B) Sagittal view of the computed tomography image demonstrating a 3.5 × 3.0-cm^2^ well-circumscribed homogeneous ovoid mass with low attenuation with a ductal remnant to the tongue base.

A 63-year-old woman presented with a painless midline neck mass for more than 3 years. She complained of gradual enlargement of the mass, leading to a lump sensation during swallowing and cosmetic embarrassment. Physical examination revealed a gross 3.5 × 3.0-cm^2^ soft midline mass located between the thyroid cartilage and hyoid bone (Fig. 1A). The mass could move upwards when the patient protruded her tongue. Sonography revealed a 3.5 × 3.0 × 3.0-cm^3^ homogenous isoechoic oval mass beyond the thyroid glands without an acoustic shadow. Ultrasound-guided core needle biopsy was performed, and histocytological analysis revealed abundant sheets of fat cells with infiltration of lymphocytes and histiocytes. Computed tomography (CT) revealed a 3.5 × 3.0 × 3.0-cm^3^ well-circumscribed homogeneous ovoid mass with Hounsfield unit (HU) between −5 and −100 and a thyroglossal duct remnant (Fig. 1B). The aforementioned findings supported the diagnosis of thyroglossal duct lipoma.

**Figure 2:**
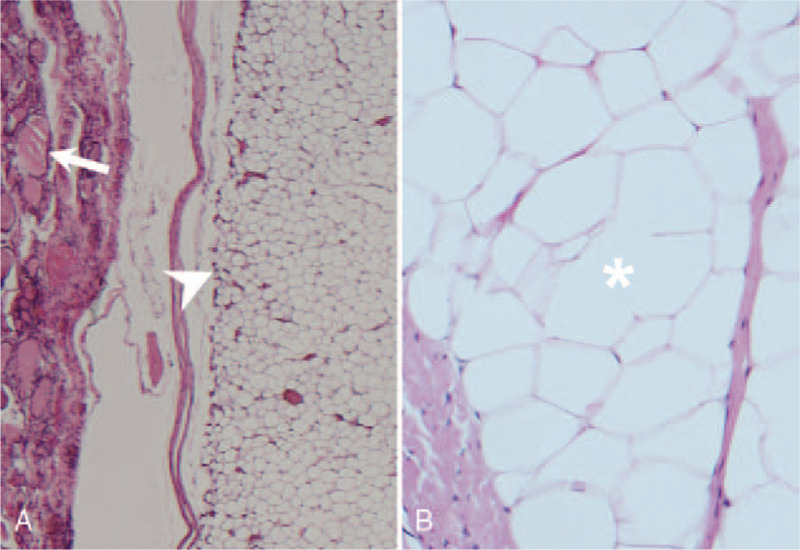
Histopathological examination of thyroglossal duct lipoma. (A) A well-defined adipose tumor (arrowhead) with a rim of benign thyroid tissue (arrow) at its periphery (hematoxylin-eosin staining, 40×). (B) High-power magnification view revealing mature adipocytes (asterisk; hematoxylin-eosin, 400×) within the adipose tumor.

The patient underwent Sistrunk operation for the excision of the midline neck mass. During surgery, a lipoma-like mass was identified with a ductal remnant under the hyoid bone, extending to the tongue base. En bloc excision of the mass with the central part of the hyoid bone and attached remnant was performed through the Sistrunk approach. No postoperative complication was observed, and the patient had an uneventful recovery. Pathological examination revealed redundant nodules of the mature adipose tumor with a rim of benign thyroid tissue at its periphery (Fig. 2A and B), establishing the diagnosis of thyroglossal duct lipoma. No recurrence was noted at the 18-month postoperative follow-up.

## 3 Discussion

More than 90% of TGDCs are diagnosed as simple cysts, but other histopathological diagnoses such as lymphadenopathy, granulomatous inflammation, lymphangioma, thyroid goiter, and mucocele have also been reported.^[1]^ Malignancy arising from TGDC has also been reported.^[1,4]^ Taimisto et al reviewed 203 patients who underwent surgery for TGDCs, and they found that 3.4% had malignant diagnoses, including papillary thyroid carcinoma in 2.9% and diffuse large B-cell lymphoma in 0.5% of patients.^[1]^ Because both benign and malignant etiologies are possible in thyroglossal duct tumors, detailed preoperative survey, meticulous surgical technique, and careful pathological review are essential for accurate diagnosis and satisfactory outcomes.

In general, sonography can be the first method for examining a central neck mass, which can enable a physician to determinate the lesion content, such as cyst or solid tumor, and the location, intra- or extra-thyroid glands, which is essential for determining the lesion origin; this is more useful than a CT scan when the lesion is small. In addition, a needle biopsy for histocytological diagnosis can be instantly and easily performed under ultrasound guidance, which can facilitate the detection of the embedded malignancy in a cystic lesion.^[1]^ In addition, a CT scan demonstrating a connection between the cystic lesion and tongue base can help confirm the diagnosis of TGDC.^[5]^ A CT scan can not only recognize the anatomical relationships between TGDC and the surrounding soft tissue, but also enable the surgeon to design a correct surgical plan, especially when the lesion is considerably large, abuts the tongue base, or requires Sistrunk operation to avoid recurrence. Comprehensive assessment of the lesion before operation might reduce surgical complications and reduce postoperative recurrence.

In our case, we found that the adipose tumor was elongated from the back of the hyoid bone. This patient underwent Sistrunk operation for the excision of the thyroglossal duct lipoma. Sistrunk operation, involving the removal of the central part of the hyoid body, provides more visibility of the entire thyroglossal duct tract for comprehensive excision and is the mainstay for successful treatment of TGDC. Sistrunk operation reduced the recurrence rate from 40% to 3% to 4% compared with the simple cyst excision method.^[1]^

The thyroglossal duct is a physiologically transient channel of the thyroid, which descends from the tongue base to the neck.^[2]^ Lipomas are benign tumors composed of adipose tissue that can manifest anywhere in the body, including the thyroid gland.^[6–8]^ Therefore, the pathogenesis observed in this case may be the development of a thyroid lipoma in the thyroglossal duct tract. However, because of the disease rarity, the definite mechanism of thyroglossal duct lipoma is still not fully understood and should be further investigated.

## 4 Conclusion

We report the first case of thyroglossal duct lipoma, extending the disease spectrum of thyroglossal duct masses. Hence, thyroglossal duct lipoma should be considered in the differential diagnosis of midline neck masses.

## Acknowledgment

The authors would like to acknowledge Wallace Academic Editing for editing this manuscript.

## Author contributions

**Conceptualization:** Ming-Shao Tsai, Geng-He Chang, Yao-Te Tsai.

**Data curation:** Ming-Shao Tsai, Geng-He Chang, Yao-Te Tsai.

**Investigation:** Yao-Te Tsai.

**Methodology:** Ming-Shao Tsai.

**Resources:** Wan-Ni Lin.

**Software:** Huei-Chieh Chuang.

**Supervision:** Huei-Chieh Chuang, Wan-Ni Lin.

**Visualization:** Huei-Chieh Chuang.

**Writing – original draft:** Ming-Shao Tsai, Geng-He Chang, Wan-Ni Lin.

**Writing – review & editing:** Yao-Te Tsai.
